# Reliability of algorithmic somatic copy number alteration detection from targeted capture data

**DOI:** 10.1093/bioinformatics/btx284

**Published:** 2017-05-04

**Authors:** Nora Rieber, Regina Bohnert, Ulrike Ziehm, Gunther Jansen

**Affiliations:** Molecular Health GmbH, Kurfürsten-Anlage 21, Heidelberg, Germany

## Abstract

**Motivation:**

Whole exome and gene panel sequencing are increasingly used for oncological diagnostics. To investigate the accuracy of SCNA detection algorithms on simulated and clinical tumor samples, the precision and sensitivity of four SCNA callers were measured using 50 simulated whole exome and 50 simulated targeted gene panel datasets, and using 119 TCGA tumor samples for which SNP array data were available.

**Results:**

On synthetic exome and panel data, VarScan2 mostly called false positives, whereas Control-FREEC was precise (>90% correct calls) at the cost of low sensitivity (<40% detected). ONCOCNV was slightly less precise on gene panel data, with similarly low sensitivity. This could be explained by low sensitivity for amplifications and high precision for deletions. Surprisingly, these results were not strongly affected by moderate tumor impurities; only contaminations with more than 60% non-cancerous cells resulted in strongly declining precision and sensitivity. On the 119 clinical samples, both Control-FREEC and CNVkit called 71.8% and 94%, respectively, of the SCNAs found by the SNP arrays, but with a considerable amount of false positives (precision 29% and 4.9%).

**Discussion:**

Whole exome and targeted gene panel methods by design limit the precision of SCNA callers, making them prone to false positives. SCNA calls cannot easily be integrated in clinical pipelines that use data from targeted capture-based sequencing. If used at all, they need to be cross-validated using orthogonal methods.

**Availability and implementation:**

Scripts are provided as supplementary information.

**Supplementary information:**

Supplementary data are available at *Bioinformatics* online.

## 1 Introduction

Although copy number variation only represents about one tenth of a percent of an individual patient’s genetic variation, it affects more base pairs than single nucleotide polymorphisms or short indels (The 1000 Genomes Project Consortium, 2015). Copy number variants (CNVs) are likely to perturb normal cellular function because they cause changes in gene dosage and expression, interrupt coding sequences, interfere with gene regulation, or create genetic chimeras (Cook and Scherer, 2008). Unsurprisingly, CNVs have been implicated in a wide spectrum of human disorders ranging from autism (Pinto *et al.*, 2010) and schizophrenia (Cook and Scherer, 2008) to epilepsy (Mefford *et al.*, 2010), Down syndrome (Korenberg *et al.*, 1994) and cancer. In cancer, deletions of tumor supressor genes or amplifications of oncogenes are considered key events in tumorigenesis and progression (Zack *et al.*, 2013), which makes them major biomarker candidates for targeted therapeutic interventions. Well-known examples include trastuzumab treatment of breast cancers with ERBB2 amplifications (Pegram *et al.*, 2004; Valero *et al.*, 2011) and crizotinib therapy of lung cancers with EML4-ALK fusions, ROS1 rearrangements, or MET amplifications (Cappuzzo *et al.*, 2009; Shaw *et al.*, 2014). Somatic copy number alteration (SCNA) identification has therefore become part of routine diagnostics in several indications.

In clinical laboratories, well-studied, recurrent SCNAs are traditionally identified using immunohistochemistry or fluorescence in situ hybridization, which are highly sensitive and specific to the queried biomarker (Soda *et al.*, 2007). Yet these molecular methods are limited to known biomarkers and are hard to scale to multiple loci. Array comparative genomic hybridization (aCGH) and SNP arrays offer high-throughput alternatives that can simultaneously search copy number aberrations at thousands of loci across the genome (Carter *et al.*, 2012; Maciejewski *et al.*, 2009). In recent years, next-generation sequencing has entered the diagnostic arena, promising a single high-throughput assay able to detect the full range of genetic alterations across the genome, across exomes, or across a predefined set of genes. Importantly, an unbiased genome-wide understanding of cancer-relevant mutations provides a more complete picture of the patient’s tumor that can be exploited to effectively match molecularly targeted drugs with the genetic characteristics of the tumor (Abrahams *et al.*, 2005; Auffray *et al.*, 2009; Ginsburg and Willard, 2009).

Despite its theoretical and practical appeal, the detection of SCNAs from massively parallel sequencing methods is far from straightforward. Ideally, SCNAs are determined from whole genome sequences with uniform coverage across all positions, yet currently such comprehensive sequencing remains beyond the means of most clinics and patients. Whole exome and targeted panel approaches offer higher coverage at a much reduced cost, but suffer from several drawbacks caused by (i) non-homogeneous coverage, (ii) unequal distribution of targeted sites across the genome, (iii) the inability to find CNV breakpoints falling outside covered regions, and, particularly for panel data, (iv) sometimes extreme GC bias (Boeva *et al.*, 2014; Dohm *et al.*, 2008) and (v) large variations in the length of the targeted regions. Tumor sequences present with their own suite of challenges, because of fluctuations in sample quality (Basik *et al.*, 2013), tumor admixture with non-cancerous cells and within-tumor clonal heterogeneity (Jacoby *et al.*, 2015).

Given these issues, a CNV caller should ideally (i) find all copy number variants in all of the sequenced genomic segments (sensitivity), (ii) reliably detect breakpoints and (iii) avoid falsely calling sequencing errors, spurious fluctuations in coverage, or other systematic biases (precision). These challenges have spurred a rich body of research that has produced a wide variety of CNV tools, only a few of which were specifically designed to deal with the vagaries of tumor genetics (Boeva *et al.*, 2011, 2012, 2014; Chiang *et al.*, 2009; Koboldt *et al.*, 2012; Talevich *et al.*, 2016). Therefore, it remains unclear if modern somatic CNV callers meet the rigorous standards required for clinical applications in precision medicine.

## 2 Approach

In this study, we therefore set out to test the sensitivity and precision of four SCNA callers on clinically realistic whole exome (WES) and targeted gene panel (panel) data. First, we use simulated datasets with known mutations (gold standard), which allows comprehensive analysis of both false positive and false negative rates. Here, we specifically test the influence of low frequency variants that can be the consequence of tumor admixture, impurity, or clonality (heterogeneity). Second, to gain more realistic insights into clinical data, we also evaluate the performance of the methods on 119 whole exome endometrial cancer datasets for which SNP array data were available as a gold standard.

## 3 Materials and methods

### 3.1 Reference genomes and simulated tumor data

The construction of realistic tumor samples consisted of several steps, each of which is explained in more detail in the next paragraphs. First, to mimic realistic human genomes, a diploid normal (control) and five tumor genome reference sequences were generated by implanting germline SNPs and somatic SNVs, germline and somatic indels and germline CNVs and tumor SCNAs. Second, to simulate the heterogeneity of tumor samples, the five tumor clones were mixed at random proportions. Finally, to simulate admixture of tumor samples with normal (control) samples, each subclone mixture was further mixed with reads sampled from the control samples. This entire process was repeated five times.

In step one, a control genome was created by implanting 3 894 338 germline SNPs and indels (Mills *et al.*, 2011; Sudmant *et al.*, 2015), 939 germline deletions and 1199 germline duplications (The 1000 Genomes Project Consortium, 2015) into the GRCh37 reference genome (Lander *et al.*, 2001). The germline CNVs were randomly picked from the Database of Genomic Variants (MacDonald *et al.*, 2014) (version 2013-07-23) and ranged in size from 52 bp to 2 191 569 bp. From this control genome, five diploid ‘pure’ tumor genomes were simulated, in total containing 16 arm-length SCNAs, 50 deletions and 50 amplifications that were randomly selected from COSMIC v71 (Forbes *et al.*, 2015) or from large cohort studies (Beroukhim *et al.*, 2010; Yang *et al.*, 2013; Zack *et al.*, 2013), as well as 30 203 known somatic SNVs and 2218 somatic indels randomly selected from the ICGC and COSMIC databases. In all simulated data, overlapping CNVs were avoided. Amplifications were introduced as tandem duplications, as these are best characterized in cancer genomes. Most (90%) of the introduced CNVs were heterozygous.

Next, read simulation was performed using Wessim (Kim *et al.*, 2013). Wessim was chosen for read simulation because it emulates the laboratory process used for targeted sequencing, including DNA shearing and *in silico* probe capture by hybridization. In addition, it reproduces known biases resulting from GC-content and fragment length variation, and platform-specific errors. Read simulations were conducted once for a whole-exome approach using the Agilent SureSelect Human All Exon kit v5 probes, and once for a targeted gene panel, using the Molecular Health Cancer Gene Panel containing 619 genes (Supplementary Material S1).

In the second step, a heterogeneous tumor sample consisting of several subclones was constructed by sampling the reads of the five pure tumor clones in different (randomly chosen) proportions of 0.27, 0.29, 0.36, 0.06 and 0.02.

In the third and last step, this clonal mixture was used to create ten admixed tumors, by adding 0% to 90% of reads from the control sample. The entire process, starting from the construction of the genome sequences, was repeated five times, such that in total we obtained 50 admixed tumor-control sample pairs with a total of 5800 SCNAs.

The final FASTQ files consisted of 101 bp Illumina paired-end reads with a mean insert size of 300 bp and 158x mean coverage for exome, and 200 bp insert size with a mean coverage of 437x for gene panel data. The datasets covered 50 390 601 and 2 383 840 nucleotides, respectively. Each dataset was aligned to the GRCh37 reference using Novoalign 3.00.03 (www.novocraft.com), and realigned around indels using GATK IndelRealigner 2.2 (McKenna *et al.*, 2010). The code for our simulations is available as Supplementary Material S2.

### 3.2 TCGA patient cases

To test the performance of SCNA callers on clinical data, we obtained exome sequencing data of 119 paired tumor-normal samples from endometrial carcinoma cases from The Cancer Genome Atlas (TCGA) project (TCGAR Network, 2013). Samples from this indication can be clustered into four classes by unsupervised hierarchical clustering, ranging from samples with few SCNAs (cluster 1) to samples with a very high number of SCNAs (cluster 4) (TCGAR Network, 2013). The 119 samples were distributed as follows: 33 samples from cluster 1; 36 from cluster 2; 13 from cluster 3; and 37 from cluster 4. For each of the exome datasets, corresponding Affymetrix Genome-Wide Human SNP Array 6.0 data was available. Only SCNAs overlapping with target regions were considered during benchmarking analysis.

### 3.3 Choice of SCNA callers

Because our focus was on the evaluation of SCNA callers in the context of cancer, we only included callers that were designed to call SCNAs from tumors, and that could deal with targeted (exome and gene panel) capture data. We included CNVkit (Talevich *et al.*, 2016) v0.7.10, Control-FREEC (Boeva *et al.*, 2012) v8.9, Varscan2 (Koboldt *et al.*, 2012) v2.4.1 and ONCOCNV (Boeva *et al.*, 2014) v6.4. CNVkit uses both targeted reads and unspecifically captured off-target reads to determine genome-wide copy number. To improve SCNA detection, it can normalize GC content, repetitive sequences, and target footprint size and spacing from pooled control samples. Control-FREEC performs genome-wide copy number normalization followed by calculation of the B-allele frequency profile, and then combines and segments that information to determine genotype status for each segment. It incorporates a control sample into the analysis for normalization, and can take into account tumor purity. ONCOCNV was specifically designed for amplicon data, and offers extensive normalization of GC content, amplicon length and other technical biases via PCA of control samples. However, it does not compute B-allele frequencies, which may affect the precision of the method in admixed data. Finally, VarScan2 is a popular SNP, indel and CNV caller that simultaneously analyzes reads from control and tumor samples, and makes calls directly from normalized read depth information. All callers were tested on the simulated data, whereas only the best performing algorithms on simulated data in the first analysis were additionally evaluated using TCGA data.

Different parameter settings were tested on the synthetic data: 13 settings for VarScan2, 11 for Control-FREEC and 8 for ONCOCNV (on panel only). Parameter optimization was not performed for CNVkit, as the approach of this tool relies on off-target reads which were not present in the simulated data. Parameters included base and mapping quality thresholds, choice of segmentation algorithm, and several tool-specific modulations. In ONCOCNV, we also tested the option to use three non-matched controls, which greatly increases precision by adding more non-cancer samples (that do not need to be from the same patient). These three non-matched controls were randomly chosen from the control samples not used to simulate that sample. A complete overview of the parameters tested for each tool is provided in Supplementary Material S3.

### 3.4 Evaluation measures

For the synthetic data, SCNAs obtained from the caller were compared with the known ‘gold standard’ variants that were implanted into the datasets. For TCGA cases, the output of the bioinformatic tools was compared with the variants determined by the SNP array, which for our purposes can be considered the ‘gold standard’.

Using sparsely and unequally distributed targeted genome regions to call copy number variants creates several technical difficulties. For example, it can lead to oversegmentation due to experimentally inherent coverage inhomogeneity, and can cause larger CNVs encompassing several target regions to be missed or falsely considered separate by the caller. Moreover, the highly variable size of SCNAs, which can range up to a full chromosome arm, renders simple interval comparisons difficult and inaccurate. Given these complexities, all comparisons between predicted and gold standard SCNAs were done using three metrics: nucleotide-based, gene-based and interval-based agreement. For the gene panel analysis, the latter was only done for genes covered in the target panel. In the manuscript we will concentrate on the nucleotide-based approach, which avoids setting an arbitrary threshold on the fraction of overlap, and also accounts for complex cases where several smaller predicted SCNAs are contained within a larger gold standard SCNA. In our experience, nucleotide-based and gene-based approaches yield similar values for sensitivity and precision, while an interval-based approach with an 80% overlap criterion leads to strongly decreased sensitivity and precision estimates.

Using the definitions listed in Table 1 of true positives (TP), predicted positives (PP) and gold standard positives (GP), sensitivity and precision were calculated for each tool, parameterization and dataset. In the manuscript, we only report the results for the default and best-performing parameter settings for each tool, and dataset; the complete results can be found as Supplementary Material S4.

**Table 1.** Overview of metrics for calculation of sensitivity and precision

Approach	Metric	Calculations
Nucleotide	TP	Number of nucleotides in intersection of predicted and gold standard intervals of the same type
	PP	Number of nucleotides in predicted intervals
	GP	Number of nucleotides in gold standard intervals
	Precision	*TP/PP*
	Sensitivity	*TP/GP*
Interval	TP	Number of predicted intervals overlapping ≥ 80% of their length with gold standard intervals of the same type
	PP	Number of predicted intervals
	True Discovery (TD)	Number of gold standard intervals covered ≥ 80% of their length by at least one predicted interval of the same type
	GP	Number of gold standard intervals
	Precision	*TP/PP*
	Sensitivity	*TD/GP*
Gene	TP	Number of genes in intersection of PP and GP
	PP	Number of genes covered ≥ 80% of their length by at least one predicted SCNA of the same type
	GP	Number of genes covered by ≥ 80% by at least one gold standard SCNA of the same type
	Precision	*TP/PP*
	Sensitivity	*TP/GP*

* The approach column refers to how gold standard and predicted (called) SCNAs were compared. TP: true positives; PP: predicted positives; GP: gold standard positives.

## 4 Results

### 4.1 Simulated data

First, we investigated the reliability of SCNA calling algorithms for whole exome and targeted gene panel data across a range of known tumor admixture levels. Simulated data offer the advantage that the exact size, position and copy number of each SCNA is known; therefore true positives, false positives and false negatives can be directly determined. Nevertheless, whether a given copy number variant is indeed identical to the one that is known to be present in the data can be determined in different ways (see methods). The interval-based estimates led to significantly decreased precision estimates across all simulated datasets, whereas the nucleotide-based and gene-based approaches yielded higher, more similar estimates of precision. For clarity and simplicity we therefore further concentrate on the nucleotide-based approach—the other measures lead to the same conclusions (see Supplementary Information S4-S5).

On the 50 exome datasets we tested VarScan2, Control-FREEC and CNVkit, and on the 50 gene panel datasets we additionally evaluated ONCOCNV, which was developed specifically for deep-targeted amplicon sequencing data. Because our simulated data did not contain off-target reads (outside of the capture regions), CNVkit was hampered in its performance, as it uniquely exploits such off-target reads to establish a genome-wide coverage baseline. For that reason CNVkit could not be fairly appraised using our synthetic data; the results are nevertheless included in Supplementary Material S4 for the sake of completeness.

**Fig. 1:**
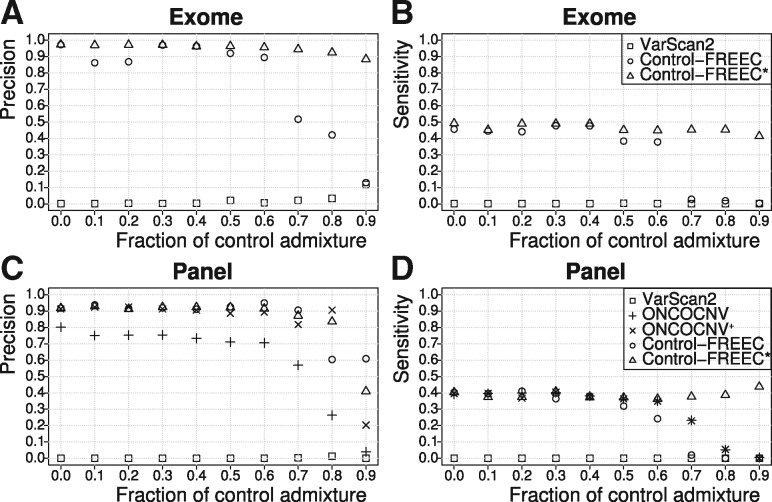
Average precision and sensitivity of SCNA callers on simulated tumors with different levels of tumor impurity. (**A**, **B**) Exome and (**C**, **D**) targeted gene panel data. Each data point represents the average of five experiments. *Control-FREEC with anticipated admixture level set as parameter; +ONCOCNV with additional pooled control samples

Across all of our experiments, VarScan2 showed surprisingly poor performance. Compared to Control-FREEC or ONCOCNV it called far more SCNAs, but most of these were false positives. Moreover, VarScan2 found almost none of the known SCNAs (Fig. 1). In contrast, Control-FREEC reached very high precision values (on average 98%) on both (non-admixed) exome and gene panel data (Fig. 1A,C). However, the method was not very sensitive, as it could only recover on average 45% of the SCNAs known to be present in the exome data (Fig. 1B), and on average 49% of the SCNAs in the panel data (Fig. 1D). ONCOCNV (on gene panel data) also produced low sensitivity (average 36.9%; Fig. 1D), but additionally had lower precision (72.6%; Fig. 1C) when using one control sample only. With pooled control samples, however, precision was similar to Control-FREEC (97%; Supplementary Material S4).

**Fig. 2:**
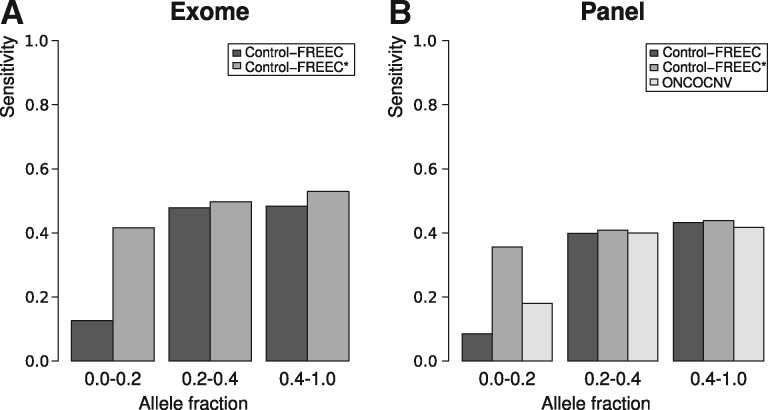
Average sensitivity of Control-FREEC and ONCOCNV in relation to SCNA allelic fraction, computed over all samples. Because the tumors were heterozygous, very few variants had an allele fraction above 0.5. Therefore all variants with an allele fraction above 0.4 were binned into one class. (**A**) Exome and (**B**) targeted gene panel data. * Control-FREEC with anticipated admixture level set as parameter. For ONCOCNV, the parameter settings did not influence the sensitivity; therefore only results for default parameters are shown

To further explain the low sensitivities of Control-FREEC and ONCOCNV, we investigated sensitivity with regard to the allele fractions at which the variants occurred in the tumors. As shown in Figure 2, the sensitivity of Control-FREEC to variants occurring at relatively high allele fractions was around 40% for both exome and panel data, but was much lower for variants with an allelic fraction below 20%. Interestingly, switching on the admixture parameter in Control-FREEC lifted the sensitivity to very low-frequency variants to the level seen for high-frequency variants, without strongly affecting the sensitivity to other variants (Fig. 2 A,B). On panel data, ONCOCNV performed similarly to Control-FREEC, except for variants in the lowest frequency class, where it outperformed default Control-FREEC, but did not score as good as Control-FREEC with the admixture parameter set.

The precision and sensitivity of ONCOCNV (on panel data) and Control-FREEC (on gene panel and exome data) were relatively robust up to 60% contamination with normal cells, but both measures dropped off steeply at higher admixtures (Fig. 1), presumably because the relative read depth difference between SCNAs and the genome background may have fallen below the detection limit. Nevertheless, when the level of admixture was provided as an input parameter to Control-FREEC, both the precision and the sensitivity of the method remained above 90% and 40% for exome, and 80% and 40% for panel, respectively. Interestingly, pooling more control samples for the ONCOCNV analysis had a similar effect (precision > 90%; sensitivity > 40%; see Supplementary Material S4).

**Fig. 3:**
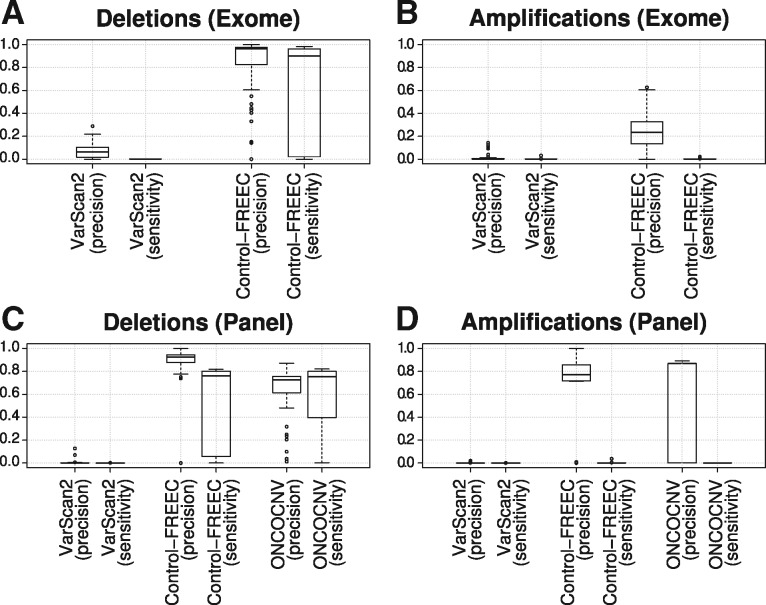
Precision and sensitivity of calling deletions and amplifications from simulated data. Each box plot is based on 50 samples. (**A**, **B**) Exome and (**C**, **D**) targeted gene panel data

Next we analyzed deletions and amplifications separately (Fig. 3; Supplementary Material S6). Both on whole exome and gene panel data, Control-FREEC reached high precision and sensitivity percentages for deletions (above 90%, except for the three highest admixture levels and for sensitivity on panel data; Fig. 3C, D;Supplementary Fig. S6). However, the results were drastically different for amplifications: on average it found less than 1% of amplifications, and about 80% of these were not present in the exome data (false positives). On panel data, precision was higher than on exome data (80%), but still with very low sensitivity (below 1%). Similarly, ONCOCNV performed relatively well on deletions (except in the most admixed tumors), but could not reliably detect amplifications. Both algorithms thus easily found deletions, but faced considerable difficulties with finding the low-frequency amplifications in our simulated tumors. Because few amplifications were called by the two algorithms, the overall high precision was mostly influenced by correctly called deletions, whereas the low sensitivity could be explained by the failure to detect amplifications.

**Fig. 4:**
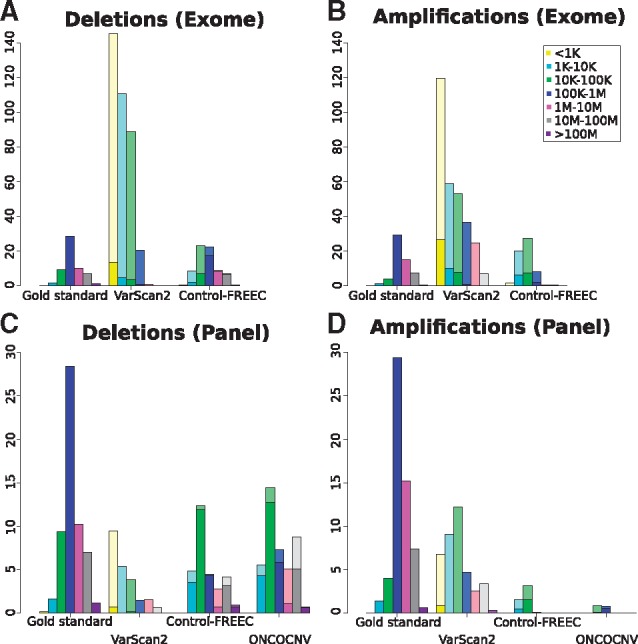
Length distributions of gold standard and predicted deletions and amplifications in **A**, **B**. simulated exome and **C**, **D**. simulated targeted gene panel data. Dark shadings indicate the number of true positives among the bioinformatically called SCNAs; the remaining (lightly shaded) calls are false positives not present in the gold standard data. The results are averaged across 50 samples. SCNAs are considered correct if they overlap for at least 80% of their length with a gold standard of the same type. To determine true positives, we only considered whether the SCNA was present in the gold standard, not whether it was called in the correct size class. Therefore the number of true positives called within particular size classes may seem counterintuitively higher than in the gold standard

Finally, we compared the length distribution of detected SCNAs across the different methods (Fig. 4). The 50 artificial genomes on average contained one or two very large (over 100 Mb) or very small (shorter than 1 Kb) SCNAs, with most SCNAs (on average 60) falling within the medium-sized class between 100 Kb and 1 Mb. Both on exome and panel data, VarScan2 called a high number of variants, with a strong bias towards the shortest lengths, most of which were false positives. On exome data, Control-FREEC mostly called short amplifications (between 1 Kb and up to 1 Mb), while the size distribution of deletions was more similar to the gold standard, but with an excess of deletion calls ranging in size between 10 and 100 Kb. The number of false positive amplifications was high for all size classes, whereas for deletions this was highest for the size classes between 1 Kb and 100 Kb. Interestingly, deletions ranging between 100 Kb and 100 Mb were mostly called correctly. On panel data, Control-FREEC almost exclusively called few short amplifications between 1 Kb and 100 Kb, whereas it correctly detected deletions across all of the size classes. False positives ranged mostly between 1 and 10 Mb. ONCOCNV found even fewer amplifications than Control-FREEC, and exclusively called amplifications ranging between 10 Kb and 1 Mb. The deletions found by ONCOCNV had a roughly similar size distribution as those called by Control-FREEC, with most false positives also in the range between 1 Mb and 100 Mb.

Taken together, these analyses show that both Control-FREEC and ONCOCNV are conservative methods with good sensitivity and precision for deletions, but limited sensitivity to low-frequency amplifications. This applied to both exome and panel data. Nevertheless, Control-FREEC and ONCOCNV generated few false positives on the simulated data, although this could be the result of the relative ‘cleanliness’ of simulated data (simulation cannot capture the full complexity of heterogeneous tumors).

### 4.2 TCGA endometrial carcinoma data

To evaluate the performance of SCNA detection algorithms on realistic clinical data, we obtained exome sequencing data of 119 endometrial cancer samples from TCGA. For these samples, SNP array data were also available as an independent proxy for ‘true’ SCNAs. The TCGA samples used in this study exhibit a wide range of SCNA numbers, and it has been established previously that the presence of SCNAs in endometrial tumors correlates with progression-free survival: most endometrioid tumors have few SCNAs, whereas serous and serous-like tumors have many (TCGAR Network, 2013). On average, the patient samples had 36 amplifications and 18 deletions in the exome capture regions, but the number of SCNAs in individual samples ranged between 0–269 amplifications and 0–100 deletions. This represents clinical reality, but it should be taken into account when interpreting the extremes in the distributions of precision and sensitivity for the TCGA dataset presented below.

**Fig. 5:**
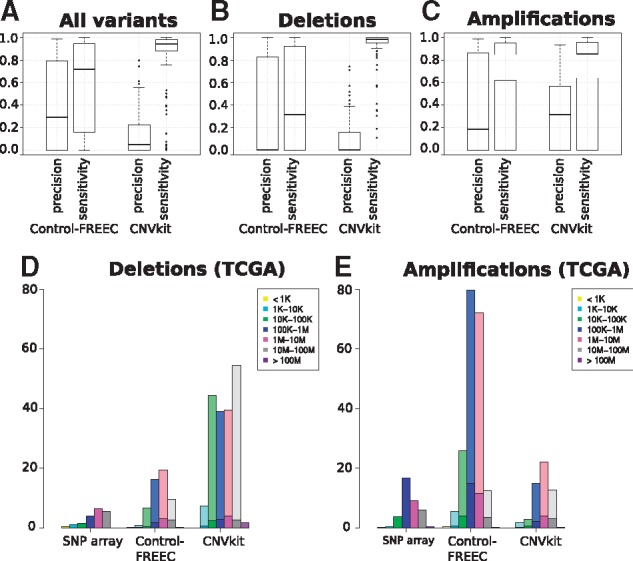
Results of SCNA calling based on 119 clinical TCGA tumor exome datasets. Precision and sensitivity for (**A**) all SCNAs, (**B**) deletions and (**C**) amplifications. Length distributions of SNP array (gold standard) and predicted (**D**) deletions and (**E**) amplifications. Dark shadings indicate the number of true positives among the called SCNAs that are also found using SNP arrays. Detected SCNAs are considered correct if they overlap over at least 80% of their length with a gold standard of the same type

Our analyses showed that Control-FREEC reached median precision of 29% and median sensitivity of 71.8% across the 119 clinical samples, although these values varied greatly depending on the patient (Fig. 5A;Supplementary Material S5). In contrast, CNVkit reached a median precision of 4.9%, but with higher median sensitivity (94%). This suggests that CNVkit called a high number of SCNAs, including both false and true positives.

Contrary to the simulation results, in half of the samples Control-FREEC detected at least 60% of the amplifications, although with many false positives (20% median precision). However, it identified few deletions (median precision < 1% with 30% median sensitivity; Fig. 5B, C). Similarly, CNVkit reached a slightly higher median precision (30%) and even over 80% median sensitivity for amplifications, but below 1% median precision and over 90% median sensitivity for deletions. Precision clearly increased with the number of somatic SCNAs present, and both precision and sensitivity were exceptionally low when few somatic SCNAs are present.

When SCNAs were separated into size classes, we observed that in each size bin Control-FREEC called many more amplifications and more deletions than were detected by the SNP arrays (Fig. 5D, E;Supplementary Material S7). However, the size distribution of the called amplifications remained similar. CNVkit showed the opposite pattern, mostly calling deletions in the medium to largest size classes, and larger amplifications ranging in size between 1 and 10 Mb. For both tools, most of the identified amplifications and deletions were not found with SNP array data.

**Fig. 6:**
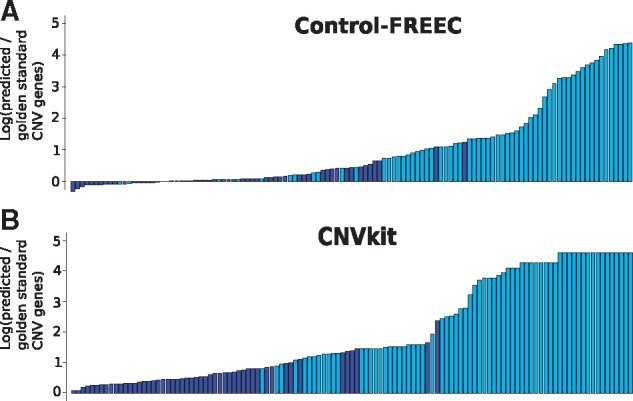
Log ratio of the number of predicted and expected copy-number-altered (gold standard) genes displayed for each individual TCGA sample. Values close to zero indicate high concordance between the number of SCNAs found by SNP arrays and the bioinformatic tools; positive values express overcalling by bioinformatic callers, and negative values suggest amplified or deleted genes found in the gold standard were not detected by the bioinformatic tools. Samples with ten or fewer SCNAs detected by SNP arrays in the exome capture regions are in light blue

To provide a better grasp of the large range in the number of calls produced on individual samples, we plotted the log ratio of predicted and gold standard copy-number-altered genes (shifting focus here to individual genes rather than SCNAs; Fig. 6). For most samples, both tools called many more copy-number-altered genes than found by the SNP arrays. This was particularly the case in samples in which fewer than ten SCNAs were detected by SNP arrays in the target regions, but for which many, sometimes thousands, of amplified or deleted genes were called. This suggests that the callers detected copy number alterations in many genes, even in samples where almost none were known to be present (unless most of the hundreds or thousands of bioinformatically called genes were missed by the SNP arrays, which we consider unlikely). This is particularly relevant considering that a small proportion of these false positive amplified or deleted genes are clinically interpretable biomarkers, and could thus mislead clinical decisions. Therefore these algorithms are insufficient to unequivocally determine whether SCNA biomarkers are present in the patient’s tumor genome.

## 5 Discussion

In this paper we aimed to evaluate the performance of several SCNA calling algorithms, including VarScan2, Control-FREEC, ONCOCNV and CNVkit, on both simulated and clinical endometrial cancer datasets. Using 50 simulated whole exome and 50 simulated targeted gene panel tumor samples, we concentrated on the ability of these methods to identify SCNAs that occur at comparatively low frequencies as a result of tumor clonality and admixture with normal, non-cancerous cells. The TCGA whole exome data allowed us to evaluate the tools in more realistic conditions, but was potentially limited by incomplete knowledge of low-frequency SCNAs in the SNP array data used as reference.

We found that both on simulated gene panel and simulated WES data, Control-FREEC produced few false positives, but could not find the majority of low-frequency amplifications. VarScan2, in contrast, produced a large number of small, spurious amplification and deletion calls. ONCOCNV slightly underperformed Control-FREEC on panel data; it discovered fewer amplifications and called more false positive deletions. However, its performance was similar when more (not necessarily matched) control samples were pooled, which enables better GC and library size normalization (Boeva *et al.*, 2014), and reduces the negative impact of germline CNVs. The results for Control-FREEC are similar to a previous study (Alkodsi *et al.*, 2015), where Control-FREEC and VarScan2 reached, respectively, 87% and 75% sensitivity on a small simulated WES dataset (based on chromosome 22 without admixture). Taken together, these results suggest that Control-FREEC performs similarly on both WES and targeted gene panel data, but on this type of data is largely incapable of discovering low-frequency SCNAs. ONCOCNV is an alternative to Control-FREEC on panel data, if provided with a sufficient number of control samples (at least three). On a side note, ONCOCNV was designed to call SCNAs from targeted amplicon sequencing, which introduces additional biases.

A different, even worse, picture emerged when we analyzed the 119 clinical endometrial carcinoma exome samples. Control-FREEC produced many more false positive amplifications and deletions on real than on simulated data, but found only 30% of deletions and only about 60% of amplifications determined by SNP arrays. CNVkit tended to correctly identify more gold standard amplifications and deletions than Control-FREEC, but at the cost of close to zero precision. Control-FREEC was biased to mostly medium-sized amplifications, while CNVkit preferentially produced medium-sized and large deletions. These data suggest that all of the tested algorithms called too many spurious SCNAs. This confirms an earlier study on 50 kidney chromophobe tumor exomes (Nam *et al.*, 2016), which reported close to zero precision, and sensitivity of 30% or 40% for Control-FREEC and VarScan2, respectively. Alkodsi *et al.* found around 30% Jaccard concordance between Control-FREEC calls and SNP arrays on four breast cancer samples (Alkodsi *et al.*, 2015). In general, our comprehensive results agree with previous studies that calling SCNAs from WES data needs to be approached with extreme caution (Alkodsi *et al.*, 2015; Guo *et al.*, 2013; Kadalayil *et al.*, 2015; Nam *et al.*, 2016; Talevich *et al.*, 2016; Tan *et al.*, 2014). A recent paper painted an even grimmer picture, confirming that even identification of structural and copy-number variation from whole genome sequencing is unsatisfactory for clinical use unless it can be supported by orthogonal technologies (Telenti *et al.*, 2016).

The discrepancy between our simulated data and the TCGA cases can be explained by several factors. First, clinical datasets feature the full biological complexities of the tumor environment and potential sample preparation and processing biases, which cannot be captured by simulations. The much-reduced precision in the TCGA data may thus be partially explained by confounding complexity, or by the presence of artifacts such as sequencing errors or differential coverage at GC-rich regions. Second, Control-FREEC had much lower sensitivity for amplifications in the simulated exome data than in the tumor samples, presumably because the real data also contained more high-copy number amplifications, which are easier to detect than duplications. On the other hand, detection of deletions was easier in the simulated than in the real data. Deletions, as an absence of signal, are expected to be easier to find. It is therefore puzzling that the precision of the tools on deletions in the TCGA data was low. Possibly this effect was negligible compared to the alltogether high number of false positives. Third, the use of SNP arrays as gold standard also introduces important limitations compared to the absolute ground truth in the simulated data. A comprehensive comparison of array-based CNV calling methods found less than 50% concordance among algorithms, and less than 70% reproducibility among replicates when the same raw data was processed multiple times with the same array platform and CNV calling algorithm (Pinto *et al.*, 2011). Moreover, SNP array profiles do not allow accurate evaluation of small CNVs that may be correctly called from WES data, and may be biased towards deletions (Pinto *et al.*, 2011). Therefore, it is quite possible that not all SCNAs that were found by the WES-based tools were false positives; at least a small portion of these may have been genuine variants not found by SNP arrays. The TCGA data unfortunately did not allow separation of true, undiscovered SCNAs from false positives, particularly at low allele fractions. Nevertheless, the high number of SCNA calls, including in samples with very few SCNAs as determined by SNP arrays, is unlikely to represent hithertho undiscovered SCNAs.

Because with simulated data we were able to rely on an absolute gold standard, we investigated how low allele fractions of SCNAs affect the sensitivity and precision of the SCNA discovery algorithms. Low allele fractions are common in clinical tumor samples, for two related reasons. First, they are caused by the presence of non-cancerous cells such as stromal cells, immune cells and blood cells present in the tumor (Fridman *et al.*, 2012; Hanahan and Weinberg, 2011), or can be a result of contamination with surrounding healthy tissue during biopsy (Basik *et al.*, 2013). These reduce the proportion of tumor-derived reads, and thus negatively impact the signal-to-noise ratio in the sample. On the other hand, it is becoming clear that tumors are not homogeneous entities consisting of clonal cells with perfect copies of the same genome, but are complex collections of distinct subclonal populations that differ both from other clones and from their non-cancerous ancestral cells (Gerlinger *et al.*, 2012; Jacoby *et al.*, 2015). Moreover, these clones are continuously subjected to local selection in the tumor environment, leading to increasing intra-tumor heterogeneity as the tumor grows (Burrell *et al.*, 2013). Some of the clones at low frequency may be important therapeutically, because they may harbor resistance mutations that can cause the eventual failure of seemingly successful chemotherapies aimed at initially dominant clones (Burrell and Swanton, 2014; Landau *et al.*, 2013). Our data suggest that up to 60% contamination with normal cells does not severely impact the performance of SCNA callers (although sensitivity is generally low, even for non-admixed tumors, and drops significantly for variants with an allele fraction below 20%). For lower tumor cellularity, it may be advantageous to obtain more control samples (ONCOCNV) or add an estimate of tumor cellularity based on e.g. a pathological report (as a parameter for Control-FREEC). However, an important caveat to our study is that we could not unequivocally answer the question whether this also applies to more realistic tumor data. The high quality standards set by TCGA for its tumor samples result in homogeneous tumor cellularity. Nevertheless, we deem it unlikely that the very low precision in the TCGA cases was predominantly caused by undetected, low-frequency SCNAs. Rather, the data suggest it is due to too many false positives. SCNA calls from next-generation sequencing data are at best error-prone hypotheses that need careful cross-validation with orthogonal methods. We therefore cannot recommend including SCNA calls based on capture NGS data into precision medicine pipelines.

## 6 Conclusion

Whole exome and targeted gene panel sequencing are increasingly finding their way into clinical practice. Their focus on a smaller and presumably more cancer-relevant part of the genome at higher coverage offers a compelling alternative to whole genome sequencing, at a much reduced price. Although these methods are primarily used in precision medicine to detect single nucleotide variants and small indels, they offer the opportunity to simultaneously call somatic copy number aberrations. This is an attractive option considering that the presence of SCNAs such as ROS or MET amplifications offers clear therapeutic options. However, our analysis of SCNA callers using 50 simulated datasets and 119 endometrial tumors clearly shows that considerable caution is needed with targeted sequencing approaches. On such data, SCNA methods are error-prone: they both missed a considerable portion of SCNAs known to be present in our test datasets, and called a large number of false positives, especially in the TCGA data. Therefore copy number aberrations called from WES or targeted gene panel sequences should be considered hypotheses that require cautious interpretation and cross-validation with more reliable methods such as FISH, PCR, or SNP arrays. In cases where SCNA discovery is explicitly desired, researchers are recommended to contemplate appropriately powerful experimental alternatives.

## Supplementary Material

Supplementary Data
