# Supplementary material for: Reliability of algorithmic somatic copy number alteration detection from targeted capture data
Source: Bioinformatics. 2017 May 4;33(18):2791–8. doi: 10.1093/bioinformatics/btx284 (PMC5870863; doi:10.1093/bioinformatics/btx284)
Supplement: Supplementary Data [file btx284_supp.zip › btx284_suppl_data/S4_prrc_parameters_measures.pdf]

Caller	Parameter	Experiment	Interval [pr, rc]	Nucleotide [pr, rc]	Gene [pr, rc]
VarScan2	par0	Exome	[0.101, 0.000]	[0.004, 0.001]	[0.006, 0.001]
VarScan2	par1	Exome	[0.102, 0.000]	[0.004, 0.001]	[0.006, 0.001]
VarScan2	par2	Exome	[0.098, 0.000]	[0.003, 0.001]	[0.006, 0.001]
VarScan2	par3	Exome	[0.100, 0.000]	[0.004, 0.001]	[0.006, 0.001]
VarScan2	par4	Exome	[0.101, 0.000]	[0.004, 0.001]	[0.006, 0.001]
VarScan2	par5	Exome	[0.126, 0.000]	[0.004, 0.001]	[0.006, 0.001]
VarScan2	par6	Exome	[0.135, 0.000]	[0.004, 0.001]	[0.006, 0.001]
VarScan2	par7	Exome	[0.102, 0.000]	[0.004, 0.001]	[0.006, 0.001]
VarScan2	par8	Exome	[0.101, 0.000]	[0.004, 0.001]	[0.006, 0.001]
VarScan2	par9	Exome	[0.102, 0.000]	[0.004, 0.001]	[0.006, 0.001]
VarScan2	par10	Exome	[0.102, 0.000]	[0.004, 0.001]	[0.006, 0.001]
VarScan2	par11	Exome	[0.074, 0.478]	[0.197, 0.478]	[0.185, 0.453]
VarScan2	par12	Exome	[0.043, 0.487]	[0.197, 0.479]	[0.186, 0.458]
VarScan2	par13	Exome	[0.072, 0.000]	[0.000, 0.000]	[0.000, 0.000]
Control-FREEC	par0	Exome	[0.482, 0.272]	[0.956, 0.414]	[0.977, 0.447]
Control-FREEC	par1	Exome	[0.468, 0.280]	[0.968, 0.427]	[0.982, 0.450]
Control-FREEC	par2	Exome	[0.478, 0.288]	[0.960, 0.484]	[0.979, 0.459]
Control-FREEC	par3	Exome	[0.459, 0.276]	[0.956, 0.435]	[0.974, 0.447]
Control-FREEC	par4	Exome	[0.459, 0.276]	[0.956, 0.435]	[0.974, 0.447]
Control-FREEC	par5	Exome	[0.440, 0.293]	[0.957, 0.473]	[0.977, 0.457]
Control-FREEC	par6	Exome	[0.483, 0.302]	[0.958, 0.481]	[0.978, 0.457]
Control-FREEC	par7	Exome	[0.514, 0.276]	[0.952, 0.414]	[0.979, 0.440]
Control-FREEC	par8	Exome	[0.477, 0.297]	[0.957, 0.473]	[0.978, 0.452]
Control-FREEC	par9	Exome	[0.442, 0.259]	[0.960, 0.448]	[0.973, 0.456]
CNVkit	par0	Exome	[0.573, 0.595]	[0.360, 0.956]	[0.395, 0.982]
VarScan2	par0	Panel	[0.025, 0.000]	[0.000, 0.000]	[0.000, 0.000]
ONCOCNV	par0	Panel	[0.695, 0.065]	[0.726, 0.369]	[0.819, 0.465]
ONCOCNV	par1	Panel	[0.769, 0.069]	[0.909, 0.366]	[0.970, 0.426]
ONCOCNV	par2	Panel	[0.336, 0.267]	[0.605, 0.402]	[0.812, 0.444]
ONCOCNV	par3	Panel	[0.346, 0.272]	[0.758, 0.402]	[0.962, 0.461]
ONCOCNV	par4	Panel	[0.690, 0.069]	[0.714, 0.358]	[0.820, 0.428]
ONCOCNV	par0b	Panel	[0.485, 0.009]	[0.235, 0.032]	[0.386, 0.054]
ONCOCNV	par1b	Panel	[0.607, 0.000]	[0.857, 0.032]	[0.948, 0.051]
ONCOCNV	par2b	Panel	[0.290, 0.026]	[0.237, 0.041]	[0.364, 0.050]
ONCOCNV	par3b	Panel	[0.345, 0.017]	[0.547, 0.041]	[0.867, 0.050]
ONCOCNV	par4b	Panel	[0.436, 0.009]	[0.214, 0.031]	[0.270, 0.033]
Control-FREEC	par0	Panel	[0.760, 0.086]	[0.924, 0.357]	[0.978, 0.481]
Control-FREEC	par1	Panel	[0.760, 0.086]	[0.924, 0.357]	[0.978, 0.481]
Control-FREEC	par2	Panel	[0.768, 0.095]	[0.919, 0.374]	[0.978, 0.484]
Control-FREEC	par3	Panel	[0.750, 0.086]	[0.922, 0.355]	[0.978, 0.442]
Control-FREEC	par4	Panel	[0.750, 0.086]	[0.922, 0.355]	[0.978, 0.442]
Control-FREEC	par5	Panel	[0.750, 0.078]	[0.928, 0.357]	[0.978, 0.477]
Control-FREEC	par6	Panel	[0.755, 0.086]	[0.922, 0.357]	[0.978, 0.481]
Control-FREEC	par7	Panel	[0.760, 0.086]	[0.924, 0.360]	[0.978, 0.481]
Control-FREEC	par8	Panel	[0.760, 0.086]	[0.924, 0.357]	[0.973, 0.459]
Control-FREEC	par9	Panel	[0.750, 0.086]	[0.917, 0.364]	[0.978, 0.486]
CNVkit	par0	Panel	[0.316, 0.500]	[0.219, 0.560]	[0.254, 0.528]
