# Supplementary material for: Reliability of algorithmic somatic copy number alteration detection from targeted capture data
Source: Bioinformatics. 2017 May 4;33(18):2791–8. doi: 10.1093/bioinformatics/btx284 (PMC5870863; doi:10.1093/bioinformatics/btx284)

Precision and sensitivity of VarScan2 and Control-FREEC on 50 simulated exome data sets

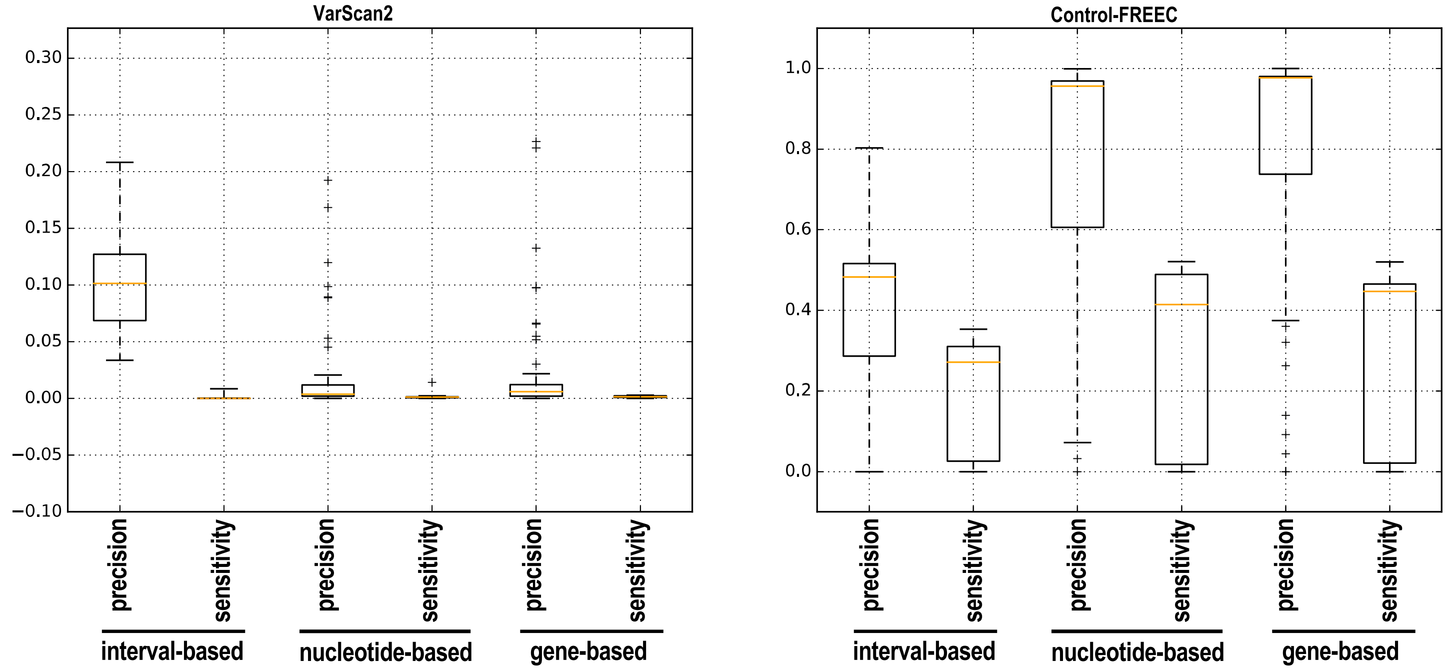

Precision and sensitivity of VarScan2, Control-FREEC and ONCOCNV on 50 simulated gene panel data sets

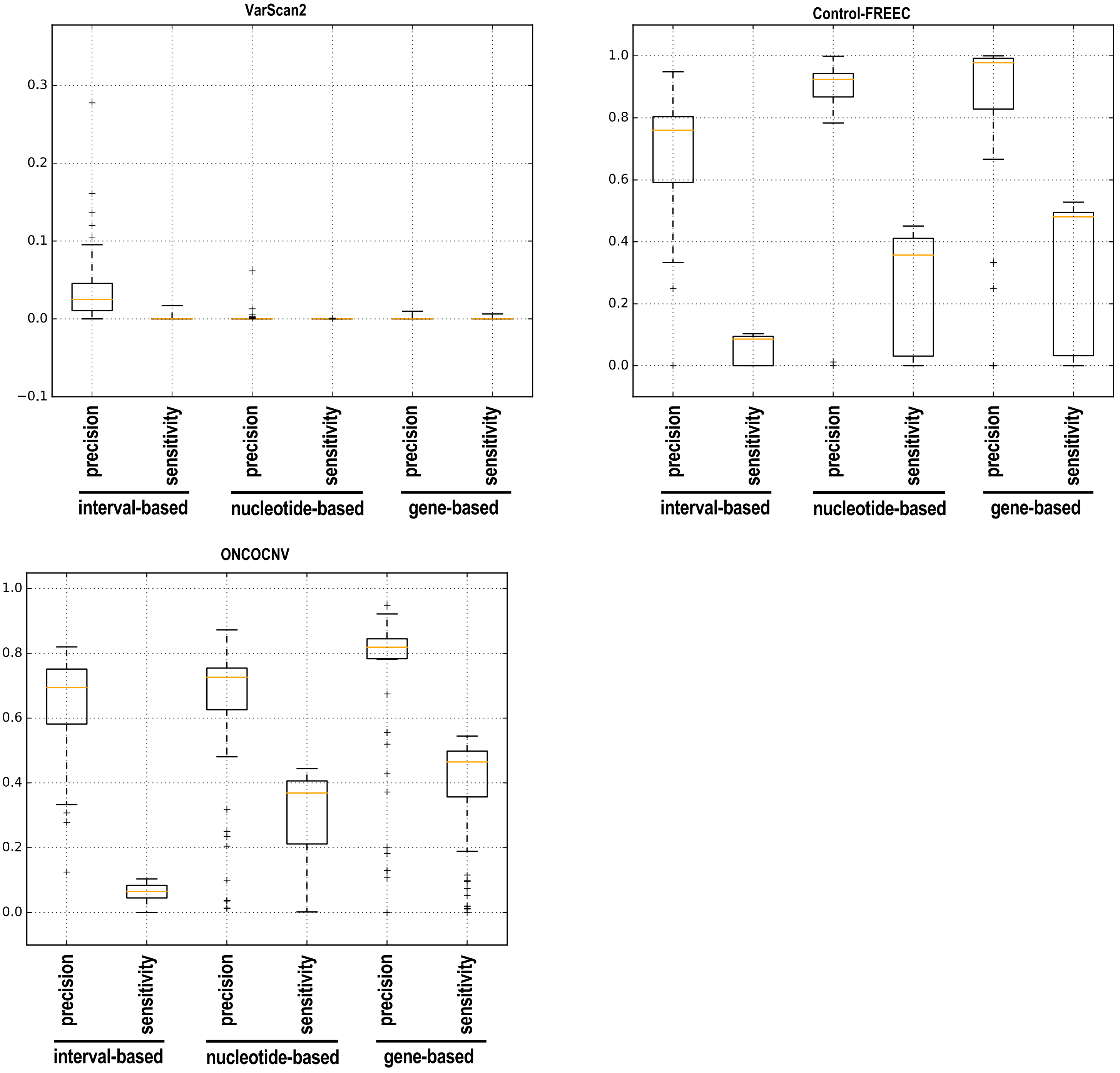

Precision and sensitivity of Control-FREEC and CNVkit on 119 TCGA tumor exome data sets

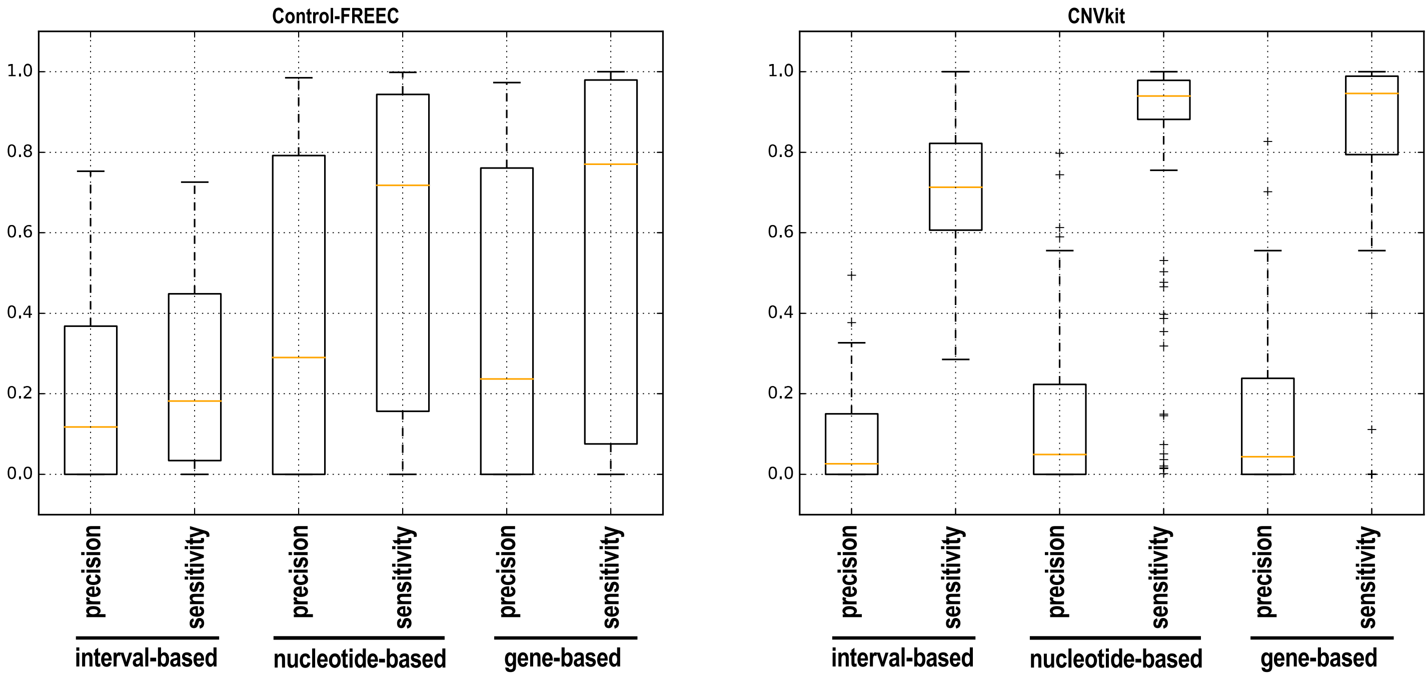

Supplement: Supplementary Data [file btx284_supp.zip › btx284_suppl_data/S5.pdf]
