# Supplementary material for: Reliability of algorithmic somatic copy number alteration detection from targeted capture data
Source: Bioinformatics. 2017 May 4;33(18):2791–8. doi: 10.1093/bioinformatics/btx284 (PMC5870863; doi:10.1093/bioinformatics/btx284)
Supplement: Supplementary Data [file btx284_supp.zip › btx284_suppl_data/S6_prrc_admixture_plot_splitbyampdel.pdf]

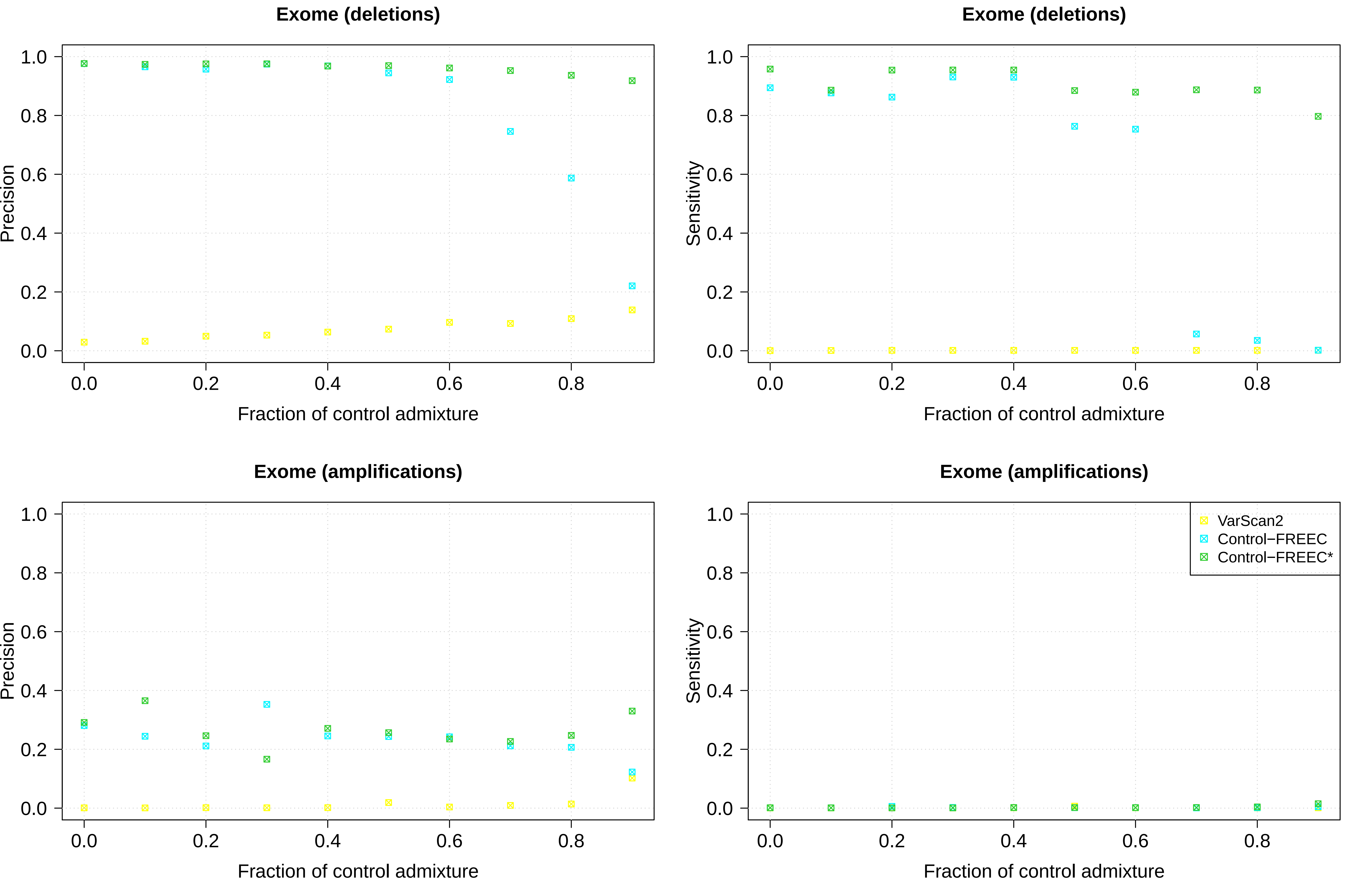

Exome (deletions)
Precision
Fraction of control admixture
Exome (deletions)
Sensitivity
Fraction of control admixture
Exome (amplifications)
Precision
Fraction of control admixture
Exome (amplifications)
Sensitivity
Fraction of control admixture
VarScan2
Control−FREEC
Control−FREEC*

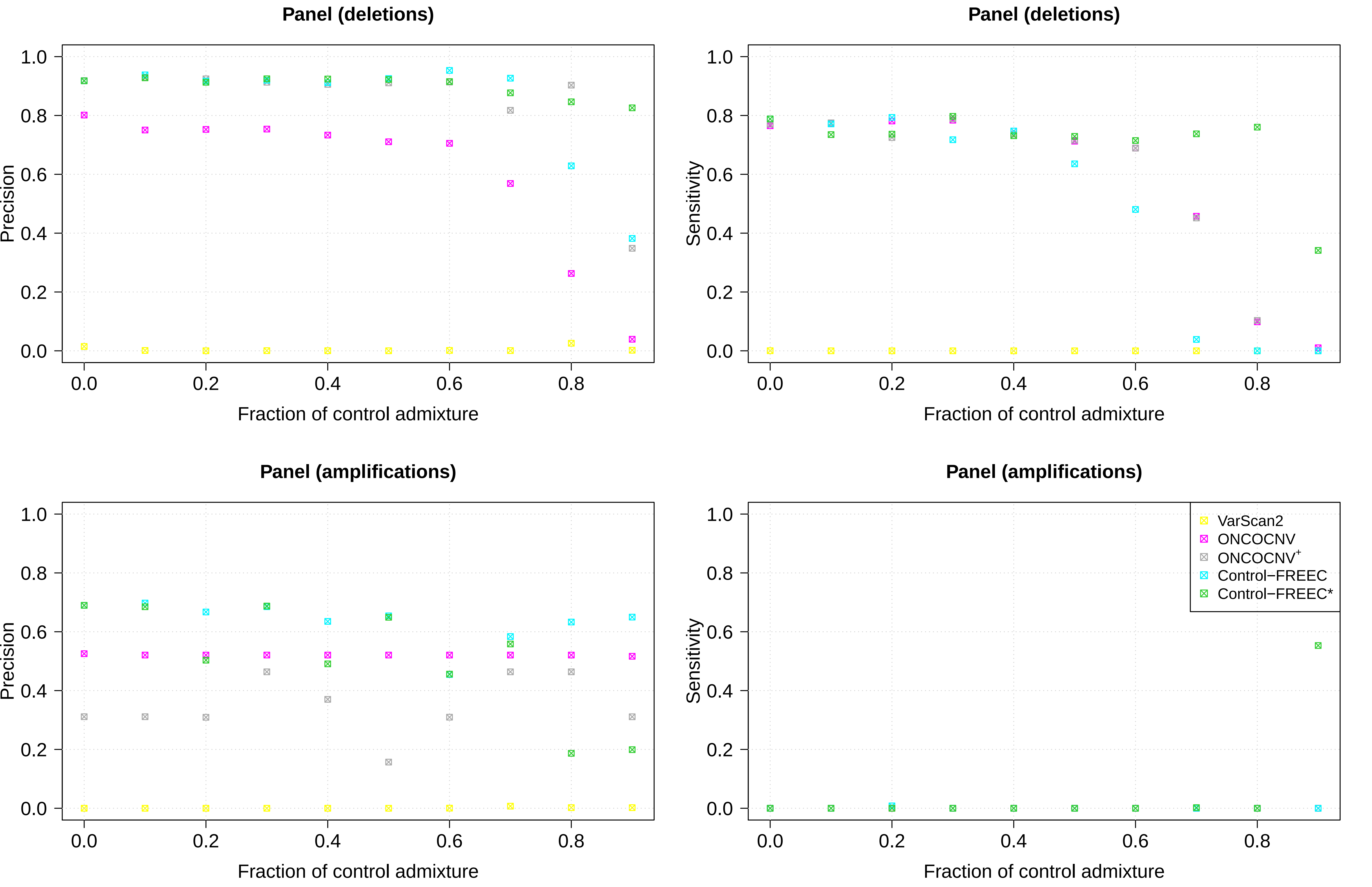
Panel (deletions)
Precision
Fraction of control admixture
Panel (deletions)
Sensitivity
Fraction of control admixture
Panel (amplifications)
Precision
Fraction of control admixture
Panel (amplifications)
Sensitivity
Fraction of control admixture
VarScan2
ONCOCNV
ONCOCNV+
Control−FREEC
Control−FREEC*
