# Supplementary material for: Reliability of algorithmic somatic copy number alteration detection from targeted capture data
Source: Bioinformatics. 2017 May 4;33(18):2791–8. doi: 10.1093/bioinformatics/btx284 (PMC5870863; doi:10.1093/bioinformatics/btx284)
Supplement: Supplementary Data [file btx284_supp.zip › btx284_suppl_data/S7_length_distro_errorbars.pdf]

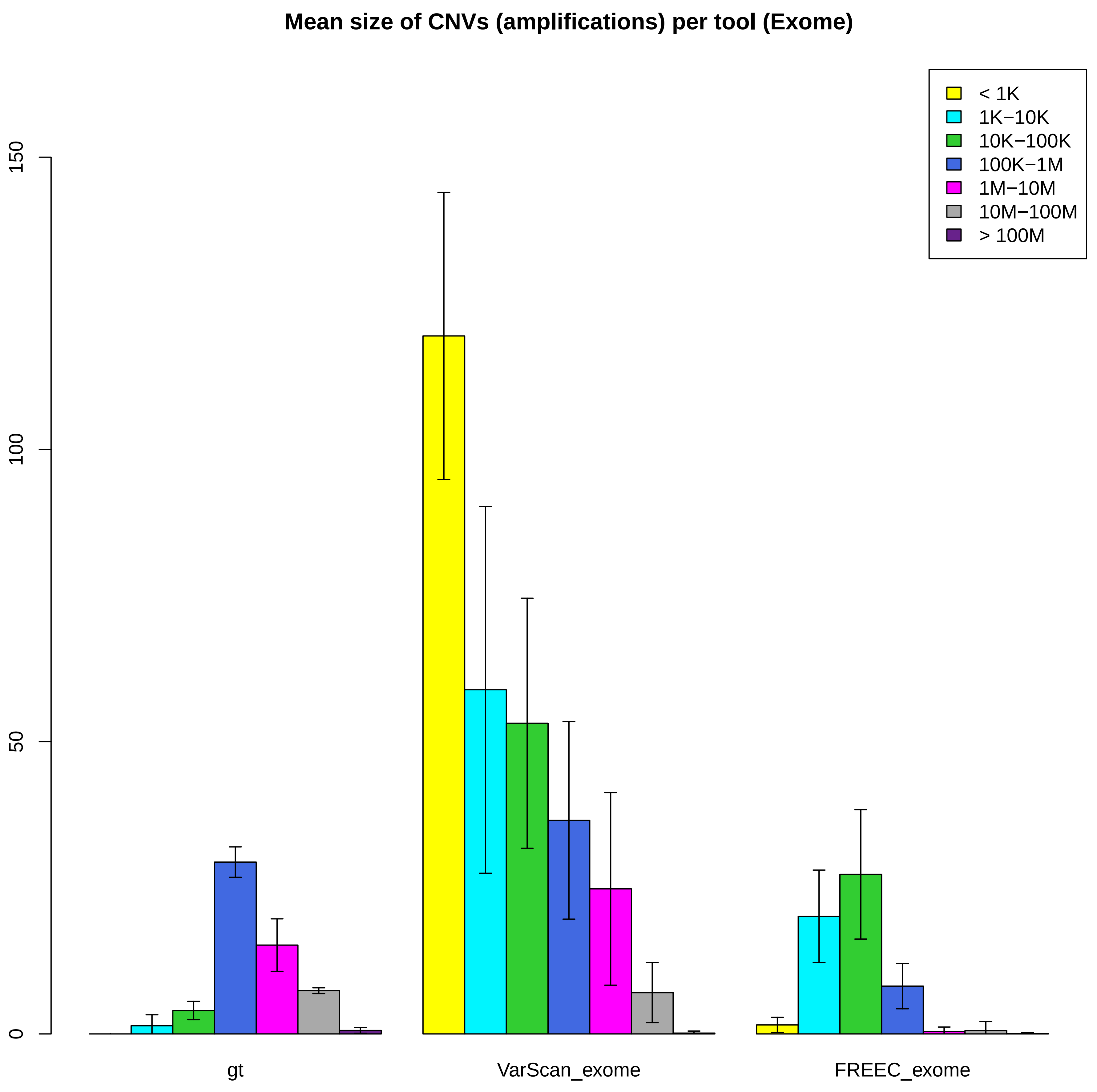
Mean size of CNVs (amplifications) per tool (Exome)
< 1K
1K−10K
10K−100K
100K−1M
1M−10M
10M−100M
> 100M
0
50
100
150
gt
VarScan_exome
FREEC_exome

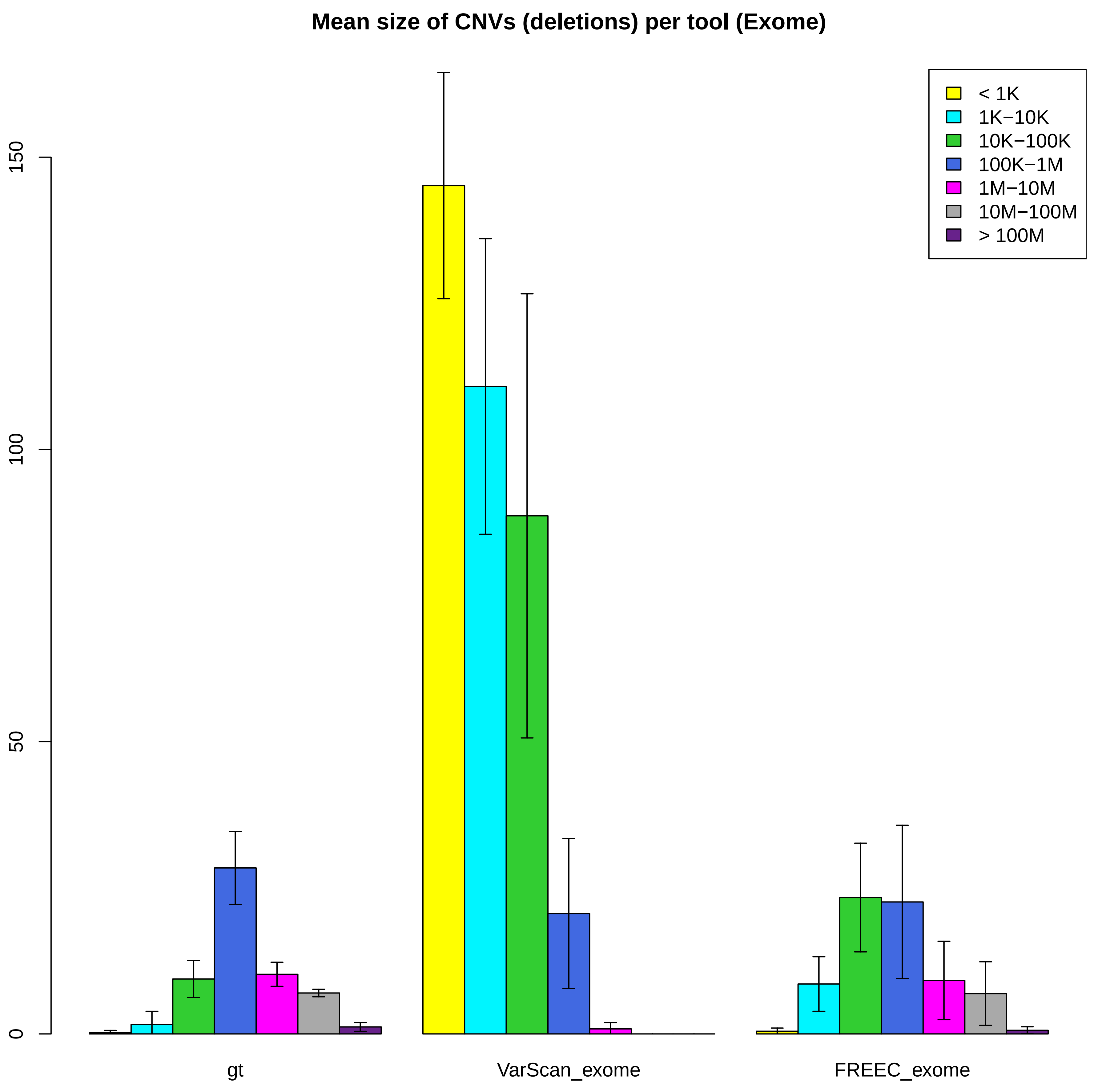
Mean size of CNVs (deletions) per tool (Exome)
< 1K
1K−10K
10K−100K
100K−1M
1M−10M
10M−100M
> 100M
0
50
100
150
gt
VarScan_exome
FREEC_exome

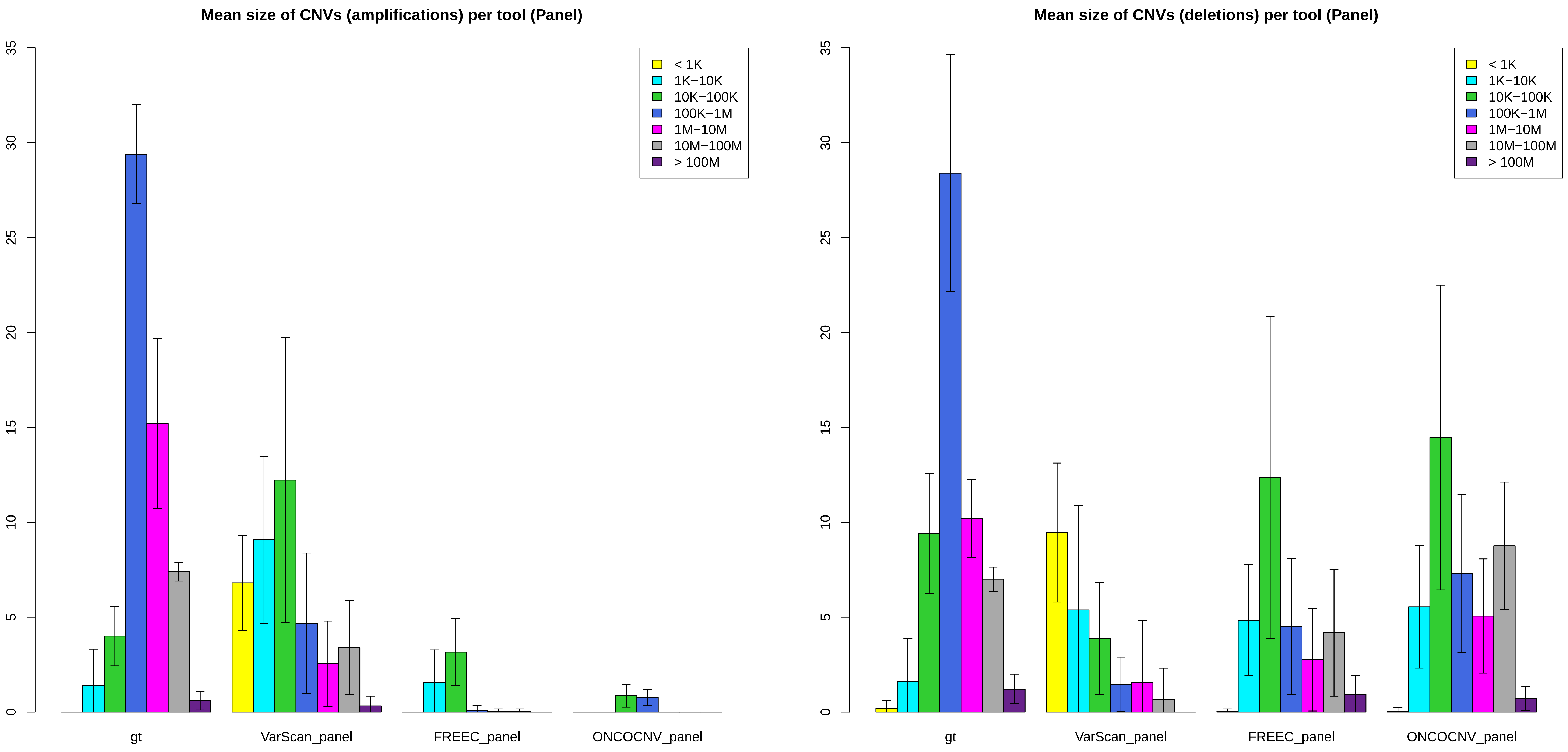

Mean size of CNVs (amplifications) per tool (Panel)
0
5
10
15
20
25
30
35
< 1K
1K−10K
10K−100K
100K−1M
1M−10M
10M−100M
> 100M
gt
VarScan_panel
FREEC_panel
ONCOCNV_panel
Mean size of CNVs (deletions) per tool (Panel)
0
5
10
15
20
25
30
35
< 1K
1K−10K
10K−100K
100K−1M
1M−10M
10M−100M
> 100M
gt
VarScan_panel
FREEC_panel
ONCOCNV_panel

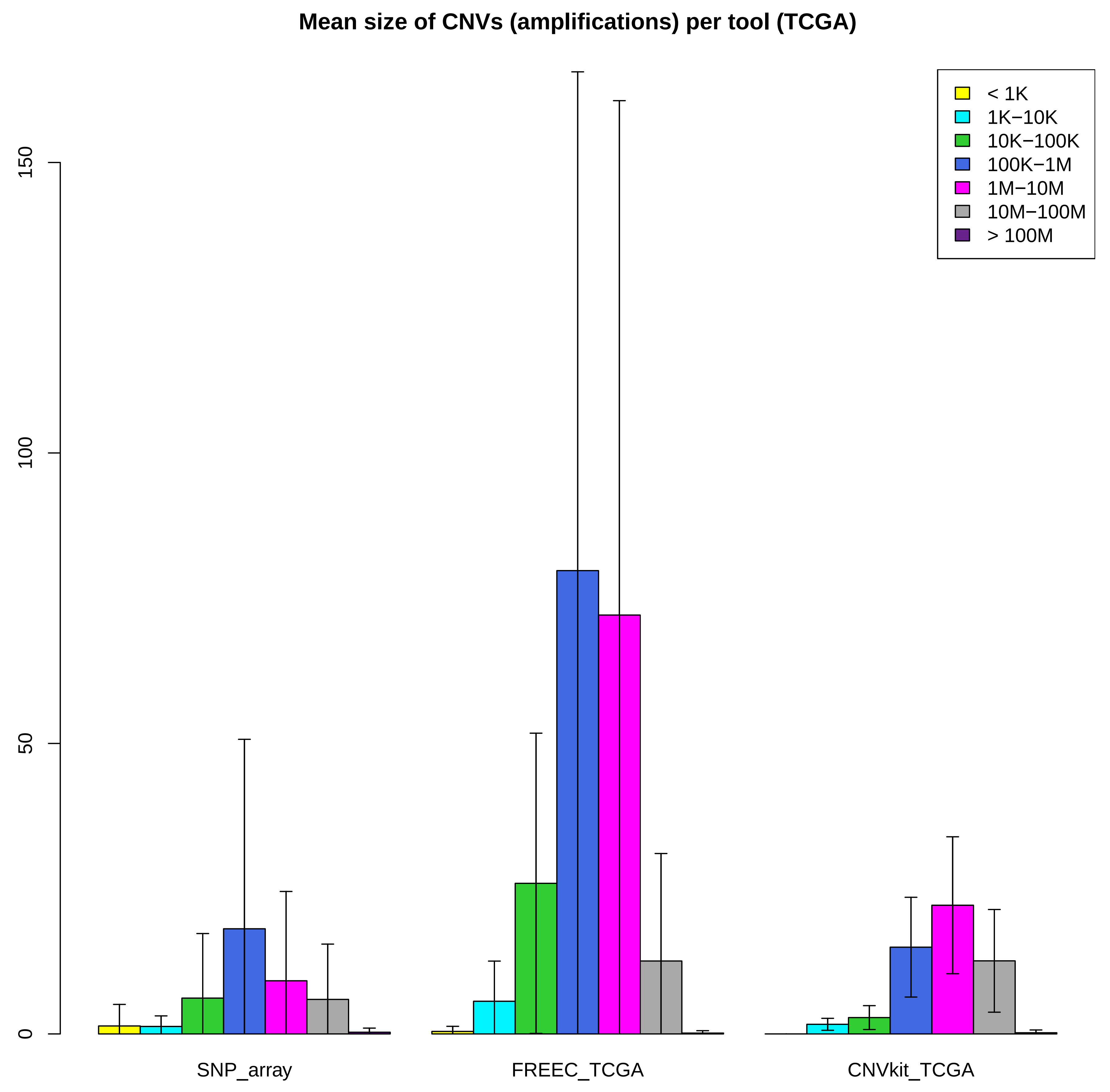
Mean size of CNVs (amplifications) per tool (TCGA)
< 1K
1K−10K
10K−100K
100K−1M
1M−10M
10M−100M
> 100M
0
50
100
150
SNP_array
FREEC_TCGA
CNVkit_TCGA

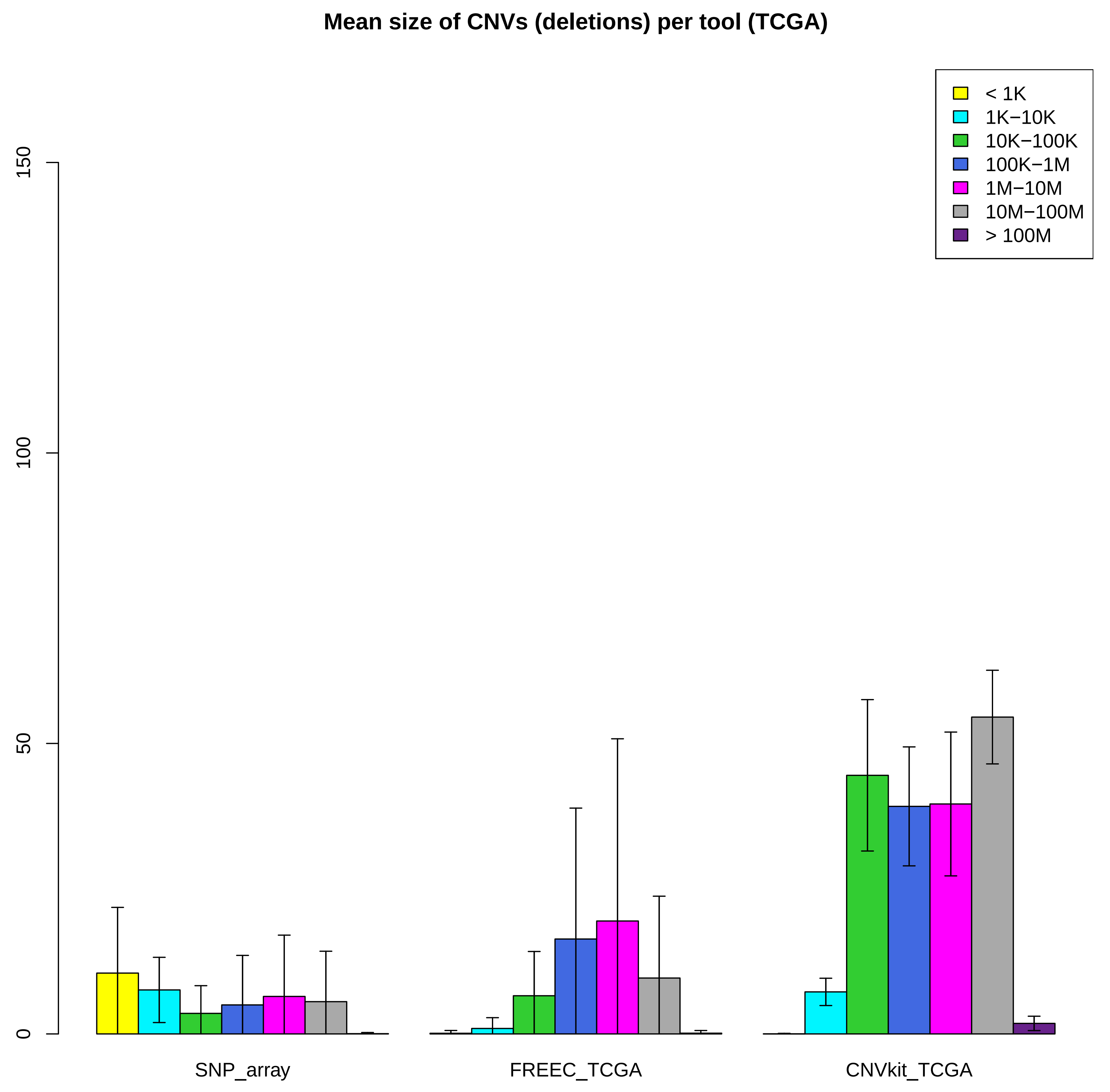
Mean size of CNVs (deletions) per tool (TCGA)
< 1K
1K−10K
10K−100K
100K−1M
1M−10M
10M−100M
> 100M
0
50
100
150
SNP_array
FREEC_TCGA
CNVkit_TCGA
